# On Luxation of the Bicuspid Teeth

**Published:** 1858-01

**Authors:** J. Fehrlin, W. G. Bennett

**Affiliations:** Surgeon Dentist, of Neufchâtel.; associate of the College of Dentists.


### ARTICLE XVII.

*On Luxation of the Bicuspid Teeth.* By M. J. Fehrlin, Surgeon Dentist, of Neufchâtel. Translated from "*L'Art Dentaire,*" by W. G. Bennett, associate of the College of Dentists.

The preservation of the teeth is so important—has so great an influence on the health—is so essential to personal beauty, and often to domestic happiness, that it is one of the great objects sought for by all conscientious dentists. The curative art of the dentist has been brought to such perfection, that he can succeed, by a rational and judicious treatment, in preserving a very great number of carious and painful teeth, with, however, the following exceptions:—1stly, those the crowns of which are too much decayed to admit of plugging, an operation to which a carious tooth should always be submitted when it is required to preserve it for any length of time. 2ndly, those attacked by periostitis, a disease which generally terminates in a fistulous opening in the gum, and in most cases, necessitates the extraction of the tooth.

It often happens that odontalgia presents itself with an intensity insupportable, produced by the exposure of the dental pulp. In such cases, the patient will not usually consent to a course of treatment which to him appears to be but the commencement of a new series of sufferings; he asks but to be relieved from agony, by the readiest possible means, the extraction of the tooth which has caused him so much pain.

It is especially in such cases when other remedies have been objected to, and when the tooth affected happens to be one of the bicuspids, that I practice luxation. This operation, which I have been in the habit of performing for nearly two years, and which, although limited in its application, has already enabled me to save a number of teeth that would have been lost without it, is performed with the key of Garengeot, and consists in raising the tooth in its socket, and replacing it immediately. The operation is somewhat analogous to that of extraction, although great difference exists between the two, it being necessary to employ much more care and precaution in making the movement for luxation than for extraction.

The result of luxation is the immediate insensibility of the dental pulp, so that the carious portion of the tooth may be removed at once, without causing the slightest irritation; the tooth is saved, and with one-half the pain to the patient than would have been caused by extraction.

Without an anatomical demonstration, one might very rationally conclude that insensibility of the dental pulp must result from the rupture of so vascular and nervous a filament as that which is broken in the process of raising a tooth in its socket. In fact, should the insensibility be caused merely by the momentary derangement of the tooth, the pain will return on the following day, or at any rate in a few days after the operation, a circumstance of rare occurrence during the two years I have practiced this treatment.

Before performing this operation, it is important to be satisfied as to the cause of the pain, for should the odontalgia be neuralgic or rheumatic, it would be better not to attempt it; otherwise, instead of affording relief, the pain would be frightfully augmented, and the tooth would have to be extracted after all. The remarks I have made respecting luxation, apply equally to extraction, which operation ought never to be performed during an attack of toothache, which is either rheumatic or neuralgic, as it would render the patient liable to increased pain after the operation.

The cases best suited to this operation are such as I have mentioned above, when the dental pulp is partially denuded of its protecting envelop, and when the pain is in consequence of this exposure. In such cases there is nothing to be feared, and success is certain, should the operation be performed with sufficient skill.

There is another case in which luxation is serviceable, that is when the dental pulp is attacked with inflammation without its being exposed to external influences. Ordinarily, the application of one or two leeches to the gum at a point corresponding with the diseased tooth will quickly cause those inflammatory symptoms to disappear, which, if neglected, are apt to result in more serious consequences. But there are persons who have the greatest dread of the application of leeches to the mouth, and who would not submit to this treatment in spite of the immediate relief afforded in cases where they are tried. It was under such circumstances a patient came to me with an upper bicuspid scarcely affected by caries, but which caused a most violent throbbing pain in the region of the diseased tooth. It was necessary that the engorged dental vessels should be relieved of their superfluous blood. The patient refused to allow leeches to be applied; without hesitation I tried luxation. Immediately after the operation the patient felt relieved; I gave a gargle of alum in cold water, and towards the end of the day the pain had nearly ceased.

The treatment after the operation consists in the employment of an emolient mouth-lotion for the first day, to be followed by an astringent one. The patient must be advised to bite gently on the affected tooth, in order to favor its fastening in the socket, and also to force out any serous fluid which might be lodged in the alveolar cavity.

As the loosening need not have been very considerable, there is no necessity to attach the tooth to its neighbors by means of a ligature. The elasticity of the walls of the alveolus will be sufficient to retain it in its place. For this purpose, it must never be forgotten to press slightly upon that part of the alveolus bent out of its position, in order that it may be placed in its original position.

Usually, from six to eight days are required to fix the tooth firmly in its socket, and nothing remains but to preserve it from the effects of caries, and to give strength and solidity to the crown, both of which effects will be produced by properly plugging the cavity.—*Ib.*

